# A SNARE-Like Protein and Biotin Are Implicated in Soybean Cyst Nematode Virulence

**DOI:** 10.1371/journal.pone.0145601

**Published:** 2015-12-29

**Authors:** Sadia Bekal, Leslie L. Domier, Biruk Gonfa, Naoufal Lakhssassi, Khalid Meksem, Kris N. Lambert

**Affiliations:** 1 Department of Crop Sciences, University of Illinois, 1102 South Goodwin Ave. Urbana, IL, 61801, United States of America; 2 Department of Plant, Soil and Agricultural Systems, 1205 Lincoln Dr. Southern Illinois University, Carbondale, IL, 62901, United States of America; INRA, FRANCE

## Abstract

Phytoparasitic nematodes that are able to infect and reproduce on plants that are considered resistant are referred to as virulent. The mechanism(s) that virulent nematodes employ to evade or suppress host plant defenses are not well understood. Here we report the use of a genetic strategy (allelic imbalance analysis) to associate single nucleotide polymorphisms (SNPs) with nematode virulence genes in *Heterodera glycines*, the soybean cyst nematode (SCN). To accomplish this analysis, a custom SCN SNP array was developed and used to genotype SCN F3-derived populations grown on resistant and susceptible soybean plants. Three SNPs reproducibly showed allele imbalances between nematodes grown on resistant and susceptible plants. Two candidate SCN virulence genes that were tightly linked to the SNPs were identified. One SCN gene encoded biotin synthase (*HgBioB*), and the other encoded a bacterial-like protein containing a putative SNARE domain (*HgSLP-1*). The two genes mapped to two different linkage groups. HgBioB contained sequence polymorphisms between avirulent and virulent nematodes. However, the gene encoding *HgSLP-1* had reduced copy number in virulent nematode populations and appears to produce multiple forms of the protein via intron retention and alternative splicing. We show that HgSLP-1 is an esophageal-gland protein that is secreted by the nematode during plant parasitism. Furthermore, in bacterial co-expression experiments, HgSLP-1 co-purified with the SCN resistance protein Rhg1 α-SNAP, suggesting that these two proteins physically interact. Collectively our data suggest that multiple SCN genes are involved in SCN virulence, and that HgSLP-1 may function as an avirulence protein and when absent it helps SCN evade host defenses.

## Introduction

The soybean cyst nematode (SCN), *Heterodera glycines*, is one of soybean’s (*Glycine max*) most damaging pathogens, causing billions of dollars in annual soybean yield losses [1]. SCN is an obligate parasite that must form a highly metabolically active, multinucleate nurse cell in the plant root (the syncytium) in order to complete its life cycle [2,3],[4,5]. The process of syncytium formation is complex and involves nematode-directed alterations of plant hormones, metabolic pathways and host gene regulation [6], in addition to suppression of host-plant defense mechanisms [7]. To prevent nematode infection, the plant utilizes several resistance mechanisms, including pre-formed defenses and specific resistance genes [8]. Understanding the mechanisms host plants use to block nematode parasitism might provide insights into how some nematodes evade these defenses.

Genetic studies have indicated that resistance to SCN is polygenic, and numerous quantitative trait loci (QTLs) for SCN resistance have been identified. However a single accession Plant Introduction (PI) 88788, and to a lesser extent soybean cultivar (cv) Peking, predominate the commercial seed market [9]. Recently, resistance genes to SCN were map-based cloned at two loci and both were shown to be atypical plant resistance genes [10,11]. The *Rhg1* locus from PI88788, was analyzed using an RNA interference (RNAi)-based approach which identified three genes that were part of a tandem repeat encoding a soybean α-SNAP protein, a wound inducible protein and a potential amino acid transporter [10]. The *Rhg4* SCN resistance gene from cv Forrest (Peking-type resistance) was recently map-based cloned using targeting induced local lesions in genomes (TILLING), in combination with gene complementation and gene silencing [11]. *Rhg4* also encoded a novel type of plant resistance gene, a serine hydroxymethyltransferase (SHMT), which is an enzyme involved in one carbon folate metabolism. The SCN resistance conferred by *Rhg4* also requires *Rhg1* to function fully, indicating the two seemingly different SCN resistance mechanisms work together in some unknown, but important way [11].

Host-plant resistance is an effective and environmentally friendly management tool. However, virulent nematode populations are able to overcome plant defenses to successfully reproduce on resistant plants. These “virulent” SCN are armed with specific virulence genes that have yet to be identified. It is suggested that nematodes probably have to overcome both innate resistance common to many plants, and induced host-plant resistance mechanisms controlled by specific nematode resistance genes [12]. In the case of basal resistance mechanisms, plant phytoalexins might be detoxified by esophageal-gland-expressed glutathione-S-transferase [13]. Likewise, an esophagus-expressed chorismate mutase (CM) is thought to play a similar role by altering the production of chorismate-derived nematode toxins [14,15]. Some SCN CM alleles showed a correlation with SCN’s ability to reproduce on some SCN-resistant soybean cultivars, suggesting some CM enzymes may aid the nematode in overcoming innate resistance mechanisms [16,17]. Other nematode effectors that have been implicated in modulating host defense are GrSPRYSEC-19 [18], Hg30C02 [19], Hs10A06 [20], Hs4F01 [21] and Mi-CRT [22].

While proteins expressed from esophageal glands are undoubtedly important for nematode parasitism, it has been also suggested that glutathione peroxidases secreted from the hypodermis could protect cyst nematodes against reactive oxygen species [23]. Likewise, a lipid binding protein secreted from the cuticle Gp-FAR-1 has been hypothesized to be an inhibitor of jasmonic acid signaling [24]. Although not secreted, biosynthetic enzymes involved in the production of vitamin B metabolites, such as pantothenate (VB5), biotin (VB7), thiamin (VB1) and pyridoxal 5-phosphate (VB6), could play a role in circumventing a starvation-based mechanism of nematode resistance [25,26]. Two avirulence genes tied to specific host plant resistance genes have been identified in root knot nematodes (*Meloidogyne spp*.); map-1protein is secreted from nematode amphids [27,28] and Mj-Cg-1 [29] which when silenced via RNAi increased the level of root-knot nematode virulence. However, the mechanism by which these genes alter nematode virulence is unknown.

In cyst nematodes the best examples of avirulence genes are the SPRYSEC effector protein Gp_RBP-1 of *Globodera pallida*, which has been shown to induce a specific hypersensitive reaction when co-expressed with the potato nematode resistance gene *Gpa-2* [30]. Likewise, the venom allergen Gr-VAP1 of *G*. *rostochiensis* triggers a cell death response in tomato (*Lycopersicon esculentum*) plants containing the Cf-2 and Rcr3^pim^ genes [31,32].

Much of how plant parasitic nematodes evade or suppress host plant resistance mechanisms depends on the corresponding plant-resistance genes involved in preventing the nematode from completing its life cycle. Thus, one might expect the unusual nematode resistance genes found at the *Rhg1* and *Rhg4* loci would require SCN to deploy equally unique mechanisms to overcome these atypical types of resistance. In this paper, we describe the use of whole genome allelic imbalance or bulk segregant analysis to identify two candidate SCN virulence genes.

## Results

### Allelic imbalance analysis

Dong & Opperman [33] established that genetic analysis of SCN virulence was feasible. The subsequent development of high throughput DNA sequencing and genotyping methods made a map-based approach to identify SCN virulence genes possible. In this project we constructed an F_3_ mapping population of SCN, segregating for virulence, and then used an allelic imbalance/bulk segregant-based approach to identify regions of the SCN genome containing virulence gene candidates.

To create the SCN mapping population, two inbred SCN strains were crossed. The female parental strain, TN10, was non-virulent and the male parental strain, TN20, was virulent on SCN resistant soybean. The resulting F_1_ nematodes were allowed to randomly inter mate for two generations to generate a mapping population of unmated F_3_ female SCN and F_3_ single cyst derived populations to use for allelic imbalance analysis.

A pool of F3 single cyst derived populations was used to inoculate soybean plants containing the *Rhg1* resistance locus, or susceptible plants harboring only the susceptible *Rhg1* alleles. The resulting cysts were harvested from all plants and the DNA was extracted. The SCN populations used in the selection experiments contained both virulent and avirulent SCN, thus one would expect the frequency of SCN virulence genes to increase in SCN populations grown on soybean harboring the *Rhg1* resistance locus, but not on the susceptible plants. Furthermore, genetic recombination in the SCN genome should create a condition in the SCN population where allelic ratios of single-nucleotide polymorphisms (SNPs) in or near SCN virulence genes should be altered, but SNPs physically farther away from or unlinked to virulence genes will not have distorted ratios of SNP alleles in comparison to the susceptible control. This type of allelic imbalance or bulk segregant analysis can be a useful genetic approach if a way of conducting high-throughput SNP analysis is available.

Since commercial genotyping tools were absent for SCN, a custom Illumina SNP array was developed. At the time of the initiation of this project, the SCN genome was not available, so cDNA sequence reads of SCN inbred strain TN10 from egg and J2 developmental stages were collected, assembled and used as a template to identify SNPs in the SCN genome. SNPs that differed between the parental strains were identified by aligning genomic sequence collected from both parental inbred strains. A total of 1536 SNPs were selected that were homozygous between the parental SCN inbred strains and these were used to generate custom genotyping oligonucleotides for the Illumina GoldenGate genotyping system.

**Fig 1:**
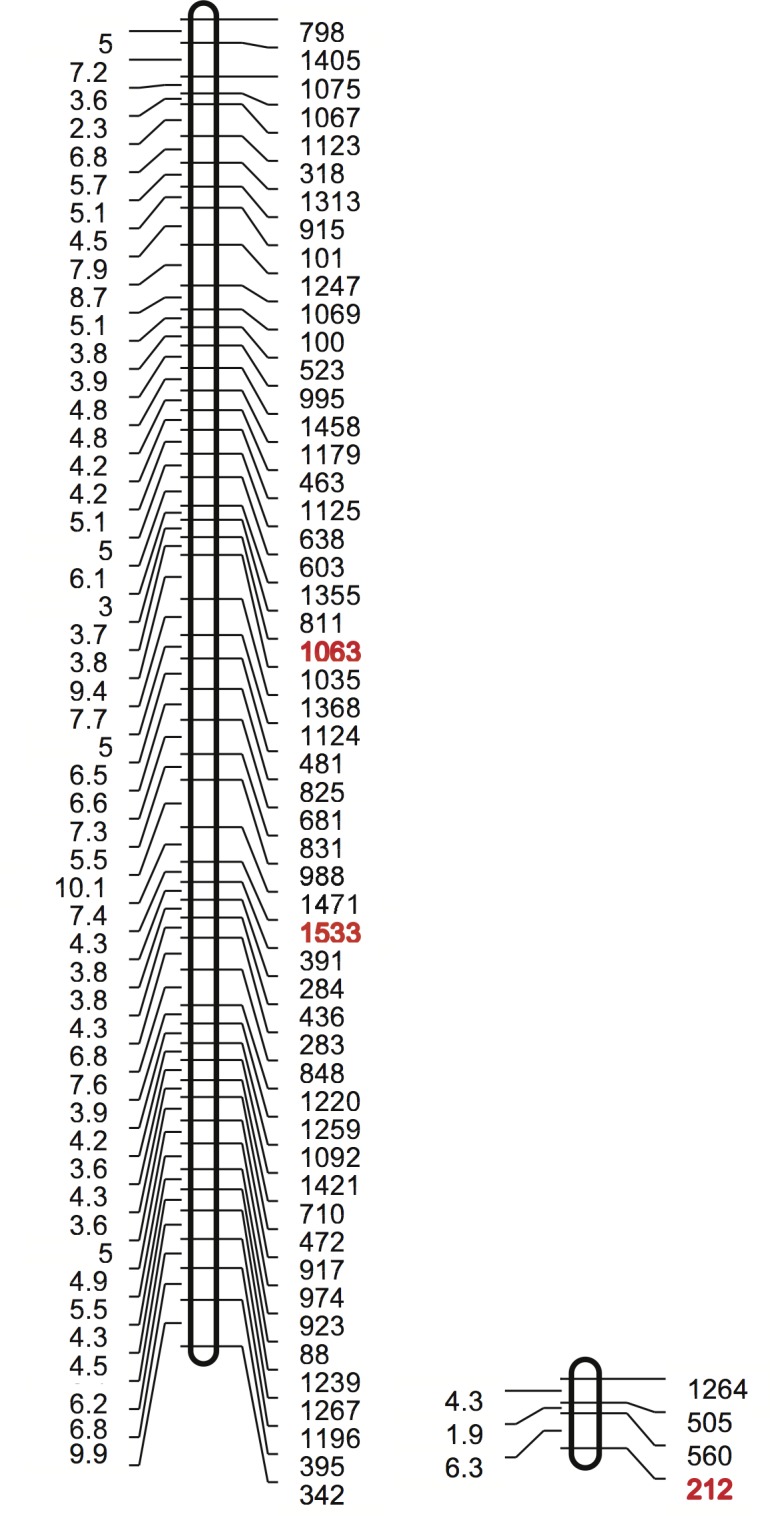
SCN genetic linkage groups containing SCN SNPs linked to virulence. The left column has the map distance in centimorgans and the right column shows the SNP number. The SCN SNPs that show an allelic imbalance when grown on resistant and susceptible soybean plants are shown in red.

Two types of SCN DNA were genotyped, one was a set of 10 DNA samples for the allelic imbalance analysis and the other was a set of 84 DNA samples extracted from the F_3_ mapping population. Parental DNA from SCN strain TN10 and TN20 were run as controls. The experiment was repeated to provide a biological replicate. In the allelic imbalance analysis, out of the 1536 SNPs tested, three SNPs showed a statically significant imbalance where the virulent and avirulent SNP allele consistently differed in frequency when the bulk SCN populations were grown on susceptible and resistant plants. Thus, the three SNPs (212, 1063 and 1533) were considered good candidates for markers linked to SCN virulence loci (Table 1). A partial genetic map containing these three SNP markers was constructed from the SCN F_3_ mapping population SNP data and showed that the three virulence associated SNPs mapped to two different linkage groups (Fig 1; S1 Table).

**Table 1:** Theta values for allelic imbalance analysis.

	SNP 212		SNP 1533		SNP 1063	
Experiment 1^a^	Susceptible	Resistant	Susceptible	Resistant	Susceptible	Resistant
Rep 1^b^	0.1894^a^	0.3439	0.5882	0.6592	0.3201	0.3737
Rep 2	0.2335	0.4187	0.607	0.6850	0.2509	0.3860
Rep 3	0.2634	0.4705	0.5903	0.6775	0.2924	0.3765
Rep 4	0.2702	0.4609	0.4251	0.7285	0.2070	0.3830
Rep 5	0.3180	0.4698	0.4775	0.6734	0.3694	0.3508
Mean	0.2549	0.4327	0.5376	0.6847	0.2879	0.374
Std Dev^d^	0.0475	0.0540	0.0813	0.0262	0.0624	0.0138
*P* ^*e*^		0.00014		0.00214		0.00243
Experiment 2						
Rep 1	0.2262	0.3309	0.4815	0.5297	0.1614	0.2084
Rep 2	0.2201	0.3086	0.4933	0.5238	0.1779	0.1796
Rep 3	0.1691	0.3076	0.4288	0.5422	0.1450	0.2192
Rep 4	0.1727	0.3484	0.3894	0.5090	0.1474	0.1812
Rep 5	0.1713	0.2608	0.4208	0.5984	0.1399	0.2389
Mean	0.1919	0.3112	0.4427	0.5406	0.1543	0.2054
Std Dev	0.0286	0.0329	0.0435	0.0344	0.0154	0.0253
*P* ^*c*^		0.00014		0.00214		0.00243

a Experiment 1 and 2 are replicates of the entire experiment

b Rep 1–5 are technical replicates within each experiment

c Significant differences in theta values for SNPs from SCN populations grown on susceptible and resistant soybean lines indicate allelic imbalances

d Std Dev = standard deviation

e P = probability from one-tailed Student's T-Test.

### Identification of linked candidate virulence genes via homology and polymorphisms

To examine the genomic regions containing the SNPs associated with virulence, a SCN genome sequence was required. Fortunately, a draft assembly of the SCN TN10 genome was recently completed by the Joint Genome Institute (JGI). BLASTN was used to match the DNA sequences flanking the SNPs to the genome scaffolds. The BLASTN search identified three scaffolds; scaffold 385 (40,259 bp) for SNP 212, scaffolds 1924 (15,316 bp) for SNP 1063 and scaffold 20 (176,619 bp) for SNP 1533.

Although it was unknown how close the SNPs might be to candidate SCN virulence gene(s), it was thought that a closely linked SCN virulence gene could be identified based upon homology to known pathogenicity related proteins and the presence of sequence polymorphisms between virulent and avirulent SCN.

Since the SCN scaffolds were from a preliminary build of the SCN genome, they were not annotated. To identify expressed genes, SCN transcriptome nucleotide sequence, derived from egg and J2 RNA, was aligned to the scaffolds. The beginning and end of the expressed genes were identified and intron sequences were removed. The resulting cDNA sequences were then compare to known proteins using BLASTX (Table 2). Plant parasitic nematodes acquire genes via horizontal gene transfer (HGT) from microorganisms [25,34], thus any HGT candidates on the scaffolds were given extra scrutiny. On two of the scaffolds, potential HGT candidates were identified. Scaffold 385 contained a gene with homology to a bacterial protein from *Paenibacillus dendritiformis*, as well as a 162 amino acid fragment of a unpublished putative dorsal esophageal gland protein, Ha-dsl-1, from *Heterodera avenae* (AD182806.1).

**Table 2:** Protein homology of expressed genes on SCN genome scaffold 385.

Seq. #	Size	Protein match	Organism	Accession #	e-Value
1	349bp	No significant similarity			
2	2822	No significant similarity			
3	787	No significant similarity			
4	1756	Phosphoglycerate mutase domain containing protein	*Haemonchus contortus*	CDJ82788.1	1e-16
5	1000	Zinc finger BED domain-containing protein	*Ciona intestinalis*	XP_004227062.1	3e-08
6	978	Protein of unknown function	*Paenibacillus dendritiformis*	WP_006677173	8e-06
7	2871	Dorsal esophageal gland protein	*Heterodera avenae*	ADI82806	6e-24
8	539	Protein of unknown function	*Haemonchus contortus*	CDJ97289	1.9e-02
9	2109	Protein of unknown function	*Ascaris*	ERG83921.1	5e-04
10	1262	No similarity			
11	2348	Protein of unknown function	*Necator americanus*	ETN70279.1	3e-09
12	2748	Regulator of nonsense transcript	*Haemonchus contortus*	CDJ83790.1	3e-12

The Ha-dsl-1 protein is 463 amino acids in length, however, only 62 amino acids of the N-terminus of the SCN Ha-dsl-1-like protein was identified in scaffold 385. The remaining 100 amino acids of the predicted SCN Ha-dsl-1-like protein were not similar to Ha-dsl-1. Furthermore, when genomic sequences from SCN TN10 and TN20, collected from an Illumina sequencer, were aligned to the SCN Ha-dsl-1-like gene, the same allelic form was present in both SCN TN10 and TN20, thus this gene was not considered a promising virulence gene candidate.

**Fig 2:**
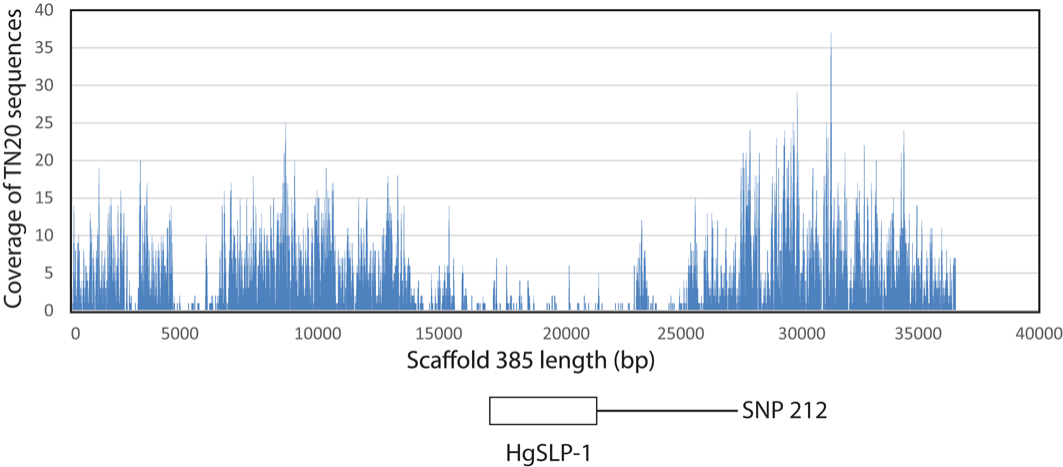
Alignment of paired SOLiD DNA sequencing reads from SCN inbred strain TN20 to the scaffold 385 reference sequence derived from TN10 genomic sequence. The Y-axis shows depth of coverage and the X-axis indicates the base position along the scaffold. The *P*. *dendritiformis*-like gene (*HgSLP-1*) spans bases 17816 to 21445.

**Fig 3:**
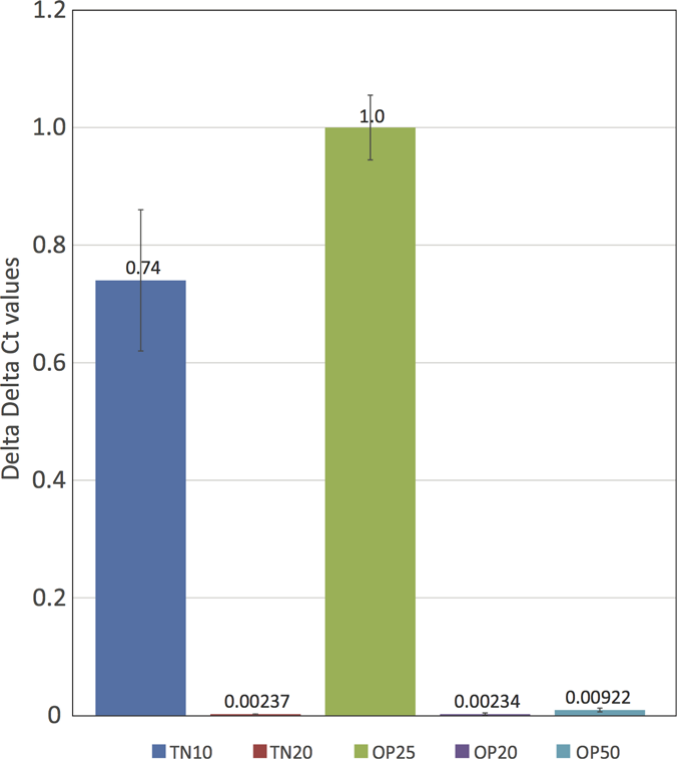
Quantitative PCR of *P*. *dendritiformis*-like gene (*HgSLP-1)* genomic copy number relative to *HgFAR-1* in inbred SCN strains, TN10, TN20 OP25, OP20 and OP50.

However, the SCN *Paenibacillus dendritiformis*-like protein in scaffold 385, which spans from base positions 18,000 to 22,000 in the scaffold, appeared more interesting. When Illumina TN10 and TN20 genomic sequences were aligned to this gene, substantially lower numbers of the TN20 sequences mapped to the SCN *P*. *dendritiformis*-like gene (Fig 2). Also, very few reads from TN20 matched a large intron spanning 4,000 to 6,000 bp, but the coding region of the gene was well covered (Fig 2). The low TN20 read coverage suggested the SCN *P*. *dendritiformis-*like gene was reduced in copy number in the virulent TN20 SCN. A TaqMan assay was developed that compared the fold difference in copy number of the *P*. *dendritiformis*-like gene to a reference SCN gene *HgFAR-1*. This assay was used to verify the copy-number reduction; an over 300-fold drop between TN10 and TN20 populations, of the SCN *P*. *dendritiformis*-like gene in the TN20 inbred SCN population (Fig 3). The copy-number reduction also occurred in two other unrelated virulent SCN strains, (OP20 and OP50) but not in another non-virulent SCN strain (OP25), suggesting the copy number of this gene in the nematode population may be important for SCN virulence (Fig 3).

SCN scaffold 1924 contained a previously identified bacterial-like biotin synthase gene (*HgBioB*) [26]. TN20 and TN10 Illumina genomic reads were aligned to the *HgBioB* gene to identify sequence polymorphisms. The HgBioB protein contained amino acid sequence differences between non-virulent TN10 and virulent TN20 SCN inbred lines at P24_A and R44_Q.

SCN scaffold 20, while larger than the others, did not contain an obvious HGT or SCN effector gene candidate.

### Gene structure and transcript variation

**Fig 4:**
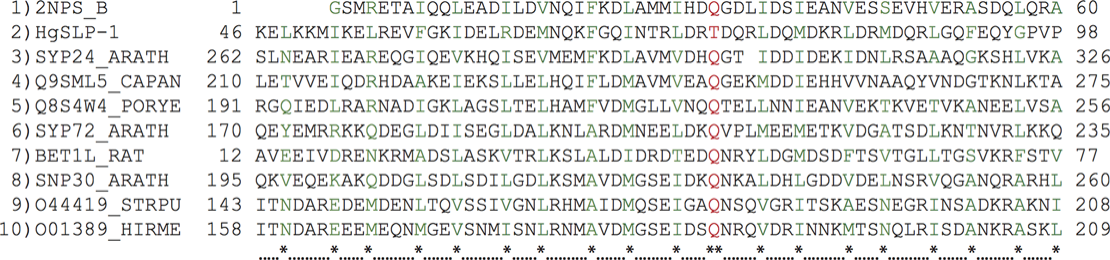
Multiple sequence alignment of the HgSLP-1 SNARE domain to related t-SNARE proteins. The **marks the zero layer residue (red) critical for membrane fusion and * indicates conserved hydrophobic residues (green) in the flanking heptad repeat domains. The following sequences are in the alignment: 1). Locus: 2NPS_B; protein name: chain B; crystal structure of the early endosomal SNARE complex; accession: 2NPS_B; organism: *Rattus norvegicus* (Norway rat). 2). Locus: HgSLP; protein name: *Heterodera glycines* SNARE-like protein 1; accession: KM575849; organism: *Heterodera glycines* (soybean cyst nematode). 3). Locus: SYP24_ARATH, protein name: putative syntaxin-24; accession: Q9C615; organism: *Arabidopsis thaliana* (thale cress). 4. Locus: Q9SML5_CAPAN; protein name: syntaxin t-SNARE; accession: Q9SML5; organism: *Capsicum annuum* (peppers). 5). Locus: Q8S4W4_PORYE; protein name: Syntaxin PM. Accession: Q8S4W4; organism: *Pyropia yezoensis* (marine red alga). 6). Locus: SYP72_ARATH; protein name: Syntaxin-72; accession: Q94KK6; organism: *A*. *thaliana*. 7). Locus: BET1L_RAT; protein name: golgi SNARE 15 kDa; accession: O35152; organism: *R*. *norvegicus*. 8). Locus: SNP30_ARATH; putative SNAP25 homologous protein SNAP30; accession: Q9LMG8; organism: *A*. *thaliana*. 9). Locus: O44419_STRPU; Protein name: Synaptosomal-associated protein 25; accession: O44419; organism: *Strongylocentrotus purpuratus* (purple sea urchin). 10). Locus: O01389_HIRME; protein name: SNAP-25 homolog; accession: O01389; organism: *Hirudo medicinalis* (medicinal leech).

The SCN *P*. *dendritiformis*-like gene in scaffold 385 was intriguing since it had the most dramatic difference between virulent and avirulent SCN in the allelic imbalance analysis, and appeared to be deleted or substantially altered in the virulent SCN parent. For this reason this sequence was chosen for further analysis. The SCN *P*. *dendritiformis*-like gene encodes a predicted protein of 326 amino acids (36.8 kDa) containing a 20-amino acid signal peptide and a 70 amino acid coiled-coil domain at amino acid positions 41 through 111. The coiled-coil region is similar to members of the target soluble *N*-ethylmaleimide sensitive fusion protein (NSF) attachment protein (SNAP) receptor domain superfamily (t-SNARE, e8.0 x 10^−3^). SNARE proteins are highly conserved in eukaryotes and are involved in mediating membrane fusion events between cell membranes. The SNARE motif consists of a central polar amino acid residue (R or Q) flanked by hydrophobic amino acids in a heptad repeat pattern that interacts with other proteins in the SNARE complex and excludes water. The SCN *P*. *dendritiformis*-like protein contained these structural motifs when compared to known plant and animal t-SNARE proteins (Fig 4), thus this sequence was named *Heterodera glycines* SNARE-like protein 1 (*HgSLP-1*). The central polar amino acid in *HgSLP-1* was threonine, which is found in some t-SNARE-like proteins that alter eukaryotic membrane fusion, but is atypical for eukaryotic SNARE motifs[35]. Since soybean SCN-resistance genes encode proteins involved in membrane fusion [10,36], this nematode gene was further characterized.

**Fig 5:**
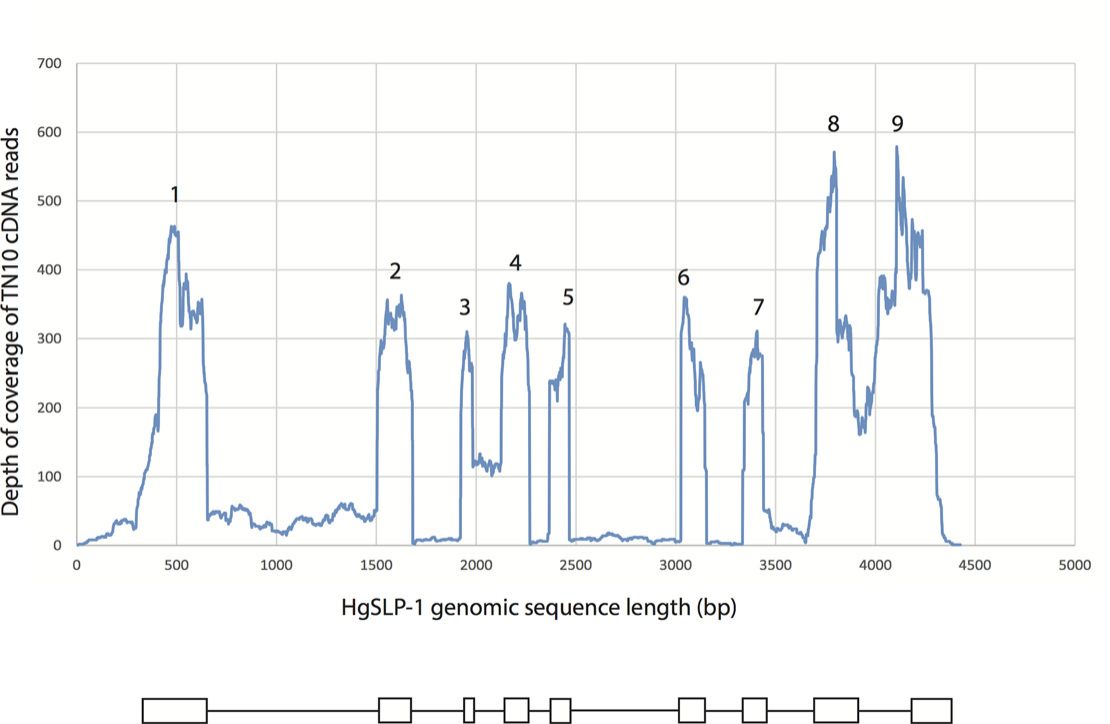
Large read mapping of TN10 cDNA sequences to the HgSLP-1 genomic sequence. The Y-axis shows depth of mapped cDNA coverage (99% identical with 99%overlap of each read) and the X-axis indicates the base position along the *HgSLP-1* gene. The numbers mark the exons of *HgSLP-1*.

The genomic sequence encoding the *HgSLP-1* gene contained 9 exons and 8 introns. However, the gene also showed evidence of intron sequence retention because most introns showed some coverage when Illumina cDNA reads were aligned to the genome sequence, with introns 3 and 8 showing the highest coverage (Fig 5). If transcripts were produced containing intron sequences, the resulting proteins would be truncated due to stop codons in all of the intron sequences. The one exception is intron 3, which does not have a stop codon, but does have enough cDNA coverage so that one third of the transcripts could retain this intron. In this case, it would be expected that a protein 48 amino acids longer would be produced (Fig 5). In addition, alignments of cDNA to the genomic sequence indicates a three-nucleotide deletion occurs 13% of the time due to an apparent alternative splice site at the beginning of exon 3. This alternative spliced form would produce a protein one amino acid shorter, missing Q107, which is at the end of the t-SNARE domain and thus could be functionally significant. Furthermore, the first exon of *HgSLP-1* appears to be similar to PTR7 and Mer40 repetitive sequences identified in SCN expressed sequence tags (ESTs) BI748250 and CB824834, respectively, making the first exon, and related sequences more abundant than a single copy gene. Most of the ESTs that matched *HgSLP-1* were only similar in the first repetitive exon, but two ESTs from SCN eggs (CB825264 and CA940412) were related to *HgSLP-1*, 73% identical over the first two exons, but only 57% identical overall. This suggests SCN expresses at least two forms of *HgSLP*, but the gene related to the EST is missing part of the t-SNARE domain, again suggesting it could have an altered function.

### HgSLP-1 protein localization

**Fig 6:**
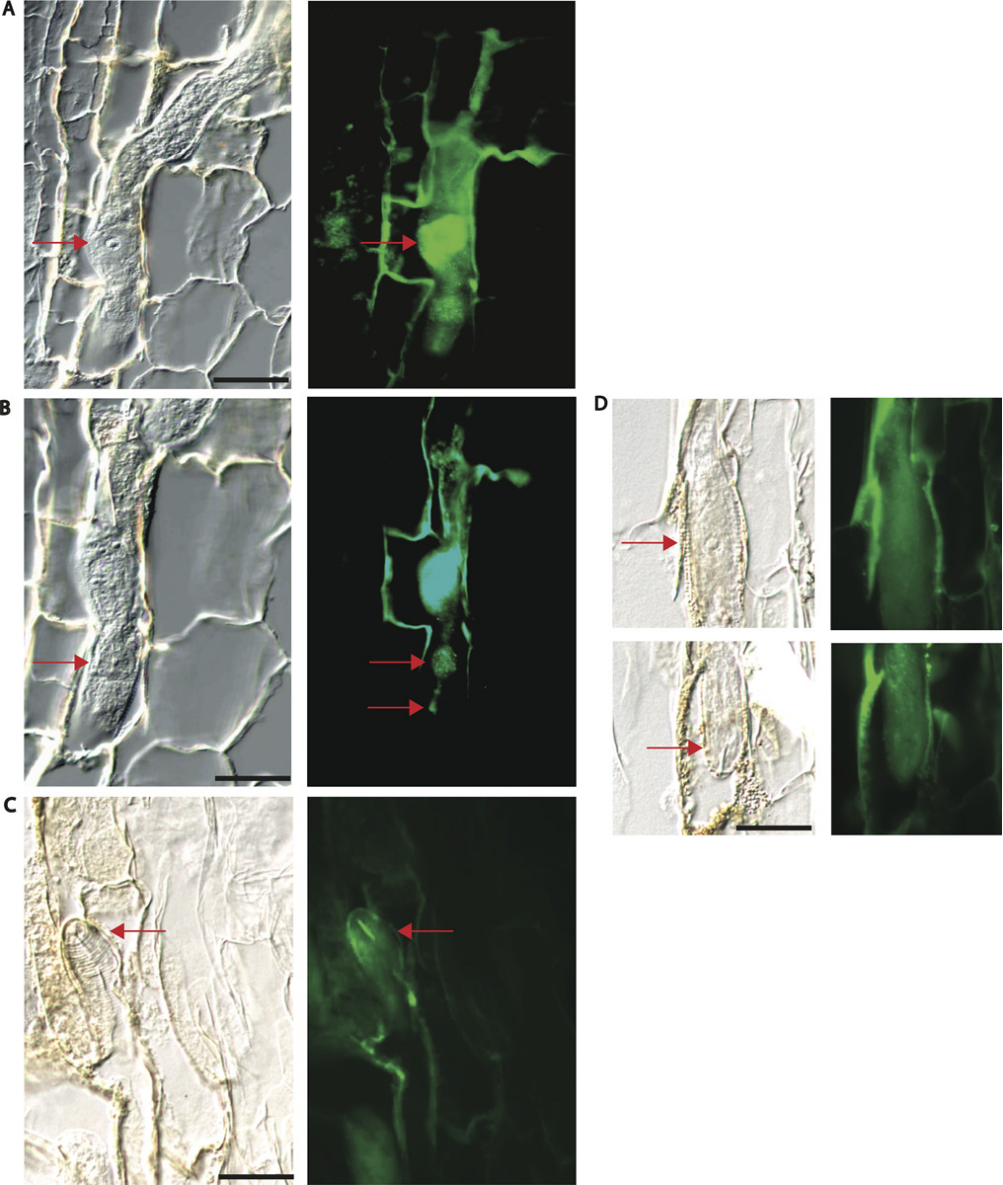
Immunolocalization of HgSNARE-like protein-1 (HgSLP-1). Panels A—D are 40 x light field images matched with corresponding epiflorescent images of sections of SCN in soybean roots stained using HgSLP-1 antibodies. Arrows point to the basal cell of a subventral esophageal gland in A, the median bulb and esophageal lumen in B and the stylet in C. Panel D shows negative control sections lacking HgSLP-1 antibody staining in the nematode. Arrows in D point to the basal cell of an esophageal gland and the stylet. Panel D is a composite of two sequential sections from the same nematode. For all light field images, 20 micron scale bars are shown.

The presence of a potential signal peptide at the N-terminus of HgSLP-1 suggested it was a possible secreted protein. Immunolocalization experiments were conducted to localize the protein in the nematode while it was parasitizing the plant to determine if it was expressed in a nematode cell type that might secrete the protein from the nematode into the plant. To do this, peptide antibodies to HgSLP-1 were produced and incubated with nematode infested root sections. When the HgSLP-1 antibodies were detected via florescent microscopy, the antibodies bound to a subventral esophageal gland, indicated by the extensive florescent signal emitted from the basal cell body (Fig 6A). Distinct antibody staining was also observed in the gland extension, metacorpus and esophageal lumen and stylet (Fig 6B and 6C). While florescent signals were also observed in plant cells walls adjacent to the nematode, we do not interpret this signal as the *in planta* location of HgSLP-1 since florescent signals are also observed in plant cell walls near the nematode in control sections. However, florescent signals in an esophageal gland or stylet were never observed in control sections. The fact that florescent signal is present in the esophageal lumen and stylet is consistent with the HgSLP-1 being secreted from the nematode since at this point the protein would have passed the valves in the metacorpus and there would be no further barriers to HgSLP-1 leaving the nematode. However, this data does not show that HgSLP-1 is injected into the nematode feeding cell. Future higher-resolution microscopic studies on SCN feeding cells will be required to address the plant subcellular location of HgSLP-1. Since HgSLP-1 appeared to be secreted, it is reasonable to assume that it could be injected into the nematode feeding cell and interact with soybean proteins.

### Characterization of HgSLP-1 by co-expression/co-purification

**Fig 7:**
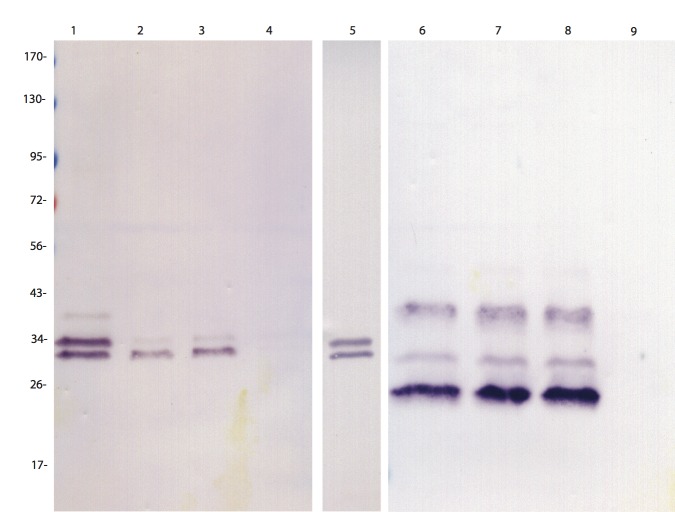
Protein gel blot of HgSNARE-like protein (HgSLP-1) and soybean α-SNAP protein expressed in *E*. *coli*. Proteins in lanes 1–5 were detected using an antibody that binds to HgSLP-1. Proteins in lanes 6–9 were detected using an antibody that binds to soybean α-SNAP. Lanes 1 and 6 contain purified protein from *E*. *coli* co-expressing full size HgSLP-1 and soybean α-SNAP. Lane 2, 3, 7, and 8 contain independent replicates of proteins purified from *E*. *coli* co-expressing HgSLP-1 missing its signal peptide and soybean α-SNAP. Lanes 4 and 9 contain purified protein from *E*. *coli* that only expresses full sized HgSLP-1. Lane 5 contains total protein from *E*. *coli* that only expresses full sized HgSLP-1. Protein sizes are shown in kDa.

The coiled-coil domain of HgSLP-1 was similar to domains found in t-SNARE proteins, suggesting that a number of plant proteins involved in membrane fusion might bind this nematode effector. Because one of the genes at the *Rhg1* locus is predicted to encode an α-SNAP, we hypothesized that the soybean *Rhg1* α-SNAP might directly bind to HgSLP-1 because α-SNAPs and t-SNARE proteins interact during the membrane fusion cycle. To test this hypothesis, we placed the genes for *HgSLP-1* and the soybean α-SNAP from the *Rhg1* locus in an *Escherichia coli* dual expression vector. For HgSLP-1 two forms were used in two different constructs, a full length and a form lacking the signal peptide. Likewise, a full-length α-SNAP gene was used, however, a C-terminal six-histidine (6xhis) tag was added to allow for purification of the expressed α-SNAP protein. The *E*. *coli* were induced to co-express both proteins and then the bacterial cells were lysed under non-denaturing conditions and the proteins purified. As a negative control we expressed just the HgSLP-1, which lacks the 6×his tag, in *E*. *coli* and attempted to purify it in parallel with the co-expressed proteins. Protein gel blots were conducted on the purified proteins and they were detected using both anti-HgSLP-1 and anti-soybean α-SNAP antibodies (Fig 7). In the lanes containing proteins purified from the co-expressed *E*. *coli*, both HgSLP-1 and soybean α-SNAP could be detected within 20 minutes, suggesting they were both abundant in the purified proteins. Trypsin digestion and mass spectrometry also detected fragments of both proteins in purified samples (data not shown). The negative control, the full size HgSLP-1 alone, did not purify, but the protein was easily detected in the initial total *E*. *coli* lysates and was equivalent in amount to the co-expressed HgSLP-1, which indicates it did not bind to the chromatography beads (Fig 7). This co-purification of HgSLP-1 and soybean α-SNAP occurred even when the metal affinity chromatography beads were very stringently washed, suggesting that the two proteins bind to each other.

**Fig 8:**
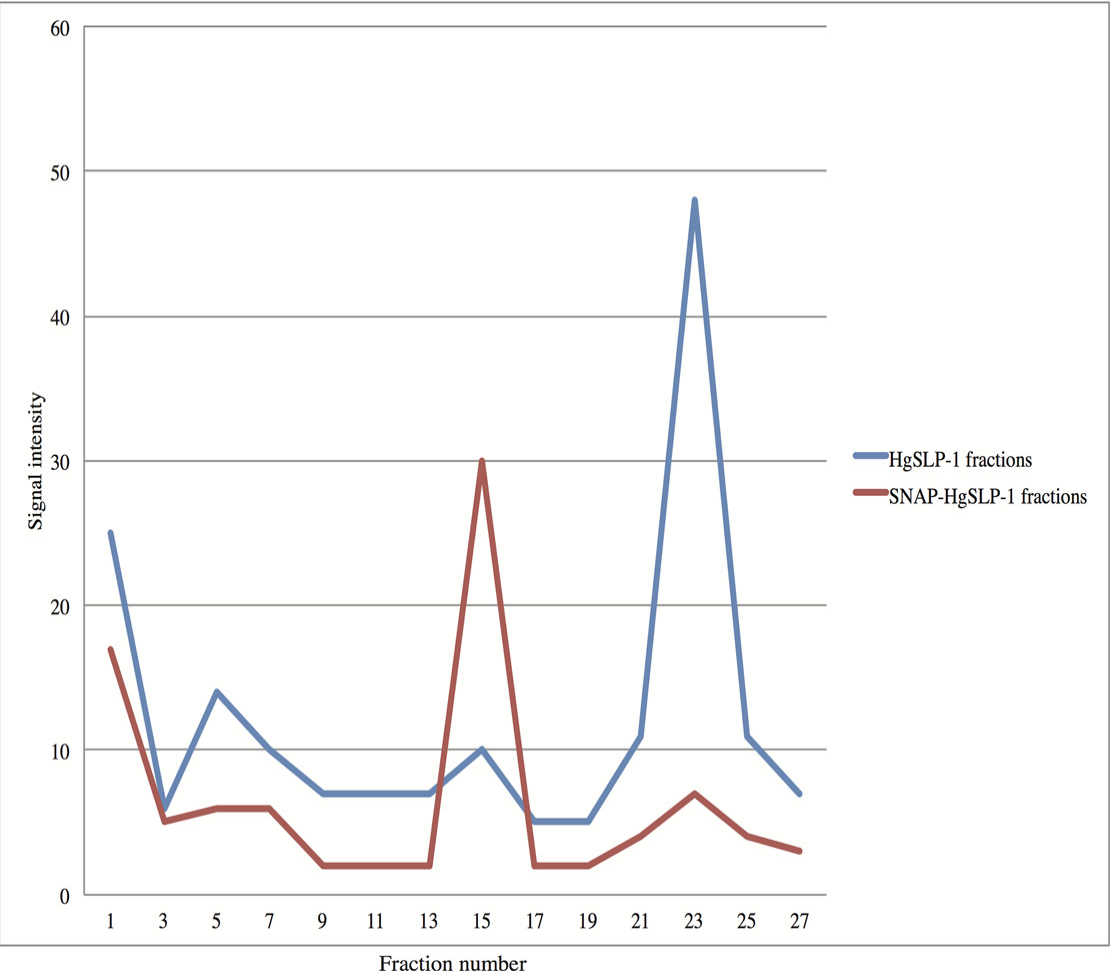
HgSLP-1 antibody binding intensity to proteins in gel filtration chromatography fractions containing either HgSLP-1 or both HgSLP-1 and α-SNAP (as indicated).

To gain additional evidence of protein-protein interaction, the total E. coli protein extracts described above containing HgSLP-1 or both HgSLP-1 and α-SNAP proteins were independently run over a gel filtration column. The fractions were collected and assayed for the presence of the HgSLP-1 protein via antibody dot blots. The HgSLP-1 (36.8 kDa) alone eluted at fraction 23 slightly sooner than the chymotrypsinogen A standard (25 kDa) which eluted at fraction 30, which is consistent with its expected size. However, when extracts containing both HgSLP-1 and α-SNAP were run through the column, the HgSLP-1 eluted at fraction 15, very close to the albumin standard (67 kDa) that eluted at fraction 12. The shift in elution of HgSLP-1 is consistent with this protein binding to α-SNAP and confirms the co-purification experiments described above. In both protein extracts, early fractions, particularly fraction 1, also contained HgSLP-1. Since fraction 1 contains proteins too large to be fractionated by the column matrix, it suggests a larger HgSLP-1: α-SNAP complex (dimer) may form (Fig 8).

## Discussion

The identification of the molecular mechanisms that plant parasitic nematodes use to evade or suppress host plant resistance is of great practical significance, since understanding this process could lead to broader and more durable resistant plants or to rapid diagnostic tests to predict the virulence profile of field nematode populations. A genetic approach to the identification of nematode virulence genes makes few assumptions about the underlying nature of the genes controlling the virulence phenotype. Past genetic studies on SCN virulence indicated one or two genes control the nematodes ability to reproduce on *Rhg1* and *Rhg4*-resistant plants [33]. This study showed that SCN was a viable genetic system; however, it was also clear that SCN lacked a genetic infrastructure (a genome sequence and sequence polymorphisms) needed for map-based cloning of the genes controlling the virulence phenotype.

Part of the problem has been resolved with the development of the semi-quantitative GoldenGate SNP assays for SCN [37] that can be used for genetic mapping and for map-based cloning via allelic imbalance [38] or bulk segregant analysis [39]. In our study, three SNPs (212, 1035 and 1533) showed consistent differences in SNP frequency when the bulk nematodes were grown on resistant and susceptible plants. Our criteria for identifying candidate virulence genes in the scaffolds of interest was based on the hypothesis that an SCN virulence gene might encode an effector protein or may have entered the genome via HGT from a microorganism and that the putative virulence gene should have a clear sequence polymorphism(s) between virulent and avirulent parents. Due to a lack of known SCN effector proteins in the SCN scaffolds under investigation, the putative HGT events in two of the scaffolds became the focus of our attention. The scaffold with SNP 1035 contained HgBioB, the scaffold with SNP 212 contained HgSLP-1, and the third SNP-containing region is still under analysis.

### HgBioB

Biotin functions as a carboxyl carrier for biotin dependent carboxylases, which are critical for fatty acid metabolism and amino acid catabolism. Biotin has also been shown to play a role in cell signaling, epigenetic regulation of genes, chromatin structure [40], and recently in microbial pathogenesis [41], making it a good candidate for a SCN virulence gene.

In general, multicellular animals, including SCN, have lost the ability to synthesize biotin *de novo*. It is assumed the gene loss occurred because animals can simply acquire the vitamins through their diet. Thus, it seems unusual that SCN, an animal and a parasite, would express biotin synthase. The discovery of *HgBioB* in one of the scaffolds associated with SCN virulence was very significant, since this gene had previously been identified and speculated to be involved in SCN virulence [26]. In fact, the SCN SNP 1035 associated with SCN virulence was in the *HgBioB* gene, making it the best candidate virulence gene in the scaffold. HgBioB has been predicted to be functional, since it retains a conserved active site, but the virulent and avirulent SCN appear to have slightly different amino acid sequences. These amino acid sequence differences could alter biotin synthase enzymatic activity and thus could be the basis of this virulence trait. The exact role HgBioB could play in virulence is unclear, but we have previously speculated it could be a method for the nematode to circumvent SCN resistance, if part of the mechanism of resistance is caused by the plant reducing biotin availability during a nematode resistance response. It should be noted, that the nematode does not have the complete biotin biosynthetic pathway, but only the last enzyme in the pathway. So, if a plant reduced biotin synthesis at the last step, as a mechanism to starve the nematode parasite, the precursors to biotin may still be available for conversion to biotin via HgBioB [25,26]. In this scenario a more enzymatically active biotin synthase enzyme, in the virulent SCN, could give the nematode a competitive advantage. It would be necessary to measure the levels of biotin in SCN feeding cells of susceptible and resistant soybean plants to test this hypothesis.

### HgSLP-1

The region of the SCN genome that contains SNP 212 encodes *HgSLP-1*, a gene that appears to have entered the SCN genome via HGT. Interestingly, *HgSLP-1* appears to vary in copy number in SCN populations. Some of the SCN strains tested for HgSLP-1 copy number variation were the genetically characterized inbred SCN strains OP20, OP25 and OP50 [33]. The genetic study of the OP SCN inbred strains established that SCN virulence was controlled by one or two genes, depending on the type of resistant plants and nematode strains used. This landmark study established that virulence on *Rhg1*-type resistance was genetically controlled by the dominant *Ror1* gene (reproduction on resistant) in the virulent strain OP50, but OP50 growth on *Rhg4*/*Rhg1*-based resistance was controlled by the recessive *ror2* gene. The virulent inbred SCN strain OP20 was shown to require two virulence genes to reproduce on the *Rhg1* source of resistance. This study suggested that SCN utilizes several mechanisms to overcome *Rhg1*-based resistance, but only one gene, *ror2*, to overcome *Rhg1/Rhg4*-type resistance. When these nematodes were tested for the presence or absence of *HgSLP-1*, the gene was present in OP25 (avirulent SCN), but nearly absent or reduced in copy number in OP20 and OP50 (both virulent on *Rhg1* and *Rhg1*/*Rhg4* resistant plants). It is interesting to note, that even though OP20, OP50 and TN20 are not related to each other (two are from North Carolina and the other from Missouri) the nematode strains have a similar virulence profile in that they grow on most SCN resistant plants. More work is needed to confirm the correlation between lower copy number of *HgSLP-1* and broad SCN virulence. However, it is interesting that the *ror2* virulence gene that controls growth on the *Rhg1*/*Rhg4*-type resistance is recessive. The deleted *HgSLP-1* gene in most of the nematodes in this strain would be expected to act as a recessive gene, thus it may be that *ror2* is in fact this deletion or lack of *HgSLP-*1.

HgSLP-1 contains a t-SNARE domain, thus it could interact with proteins involved in membrane fusion. In general, to initiate membrane fusion between a vesicle and a target membrane, a complex of proteins must interact; a vesicle will have a membrane bound v-SNARE (also referred to as R-SAREs, Vamp or synaptobrevin proteins), while the target membrane will have a different membrane bound t-SNARE protein (also referred to as Q-SAREs or syntaxin proteins) [42]. The complex also contains a protein called SNAP-25 that binds to the other SNAREs to form a stable trans-SNARE complex where coiled-coil domains bind these proteins together [42,43]. The soluble protein α-SNAP is an adapter protein and binds to the t-SNARE, NSF and to a lesser extent SNAP-25 [44] and stimulates the NSF ATPase to disassemble the cis-SNARE complex after membrane fusion [45].

The SCN gene encoding *HgSLP-1* is homologous to a bacterial protein from *P*. *dendritiformis*. A subset of bacterial SNARE proteins act as virulence effectors in intracellular human pathogens such as *Chlamydia*, *Mycobacterium*, *Salmonella* and *Legionella* [46]. In these intracellular bacteria, the SNARE domain proteins acts as a mimic and directly bind to host v-SNAREs to suppresses defense-related membrane fusion events, which aids the bacteria by preventing the phagosome membranes from fusing with lysosomes and killing the bacteria [47].

Likewise, the α-SNAP proteins can bind to t-SNAREs via coiled-coil domains both in trans-SNARE complexes and independently [48]. When α-SNAP binds to free t-SNAREs in a cell, this interaction prevents membrane fusion events [49]. Thus, it seemed possible that if the HgSLP-1 could act as a t-SNARE mimic and bind to an α-SNAP, or a soybean v-SNARE, it might prevent defense-related membrane fusion events in the nematode feeding cell by sequestering these required plant membrane fusion proteins. However in *Rhg1*-mediated resistance, plants have up to 10 copies of *Rhg1* α-SNAP, which may allow them to overcome this putative sequestration. While the data indicates HgSLP-1 is secreted from the nematode and interacts with a soybean α-SNAP, further work will be required to experimentally demonstrate that HgSLP-1 is injected into the feeding cell and interacts *in planta* with host proteins.

The role of plant membrane fusion proteins in SCN-resistance is supported by the finding that one of the *Rhg1* resistance genes encodes a α-SNAP protein. Rhg1 α-SNAP proteins may confer SCN resistance by themselves [36,50] or in combination with the other resistance genes at the *Rhg1* locus [10]. Exactly how HgSLP-1 might mitigate host defenses in soybean lacking the *Rhg-1* loci is unclear, but over expression of α-SNAP can induce syntaxin 31 in soybean and this t-SNARE protein enhances SCN resistance [50], thus blocking this interaction may promote susceptibility. It has also been demonstrated in tobacco (*Nicotiana tabacum*) that by blocking syntaxin SYP132, a t-SNARE protein involved with targeting vesicles containing PR proteins, increased bacterial susceptibility [51]. Similarly, resistance to *Peronospora parasitica* is mediated via VAMP721/722 vesicles in *Arabidopsis* [52], suggesting vesicle transport is a critical component for host-plant resistance to several plant pathogens. Thus, it seems plausible that HgSLP-1 could block the vesicle-mediated transport of anti-nematode proteins or metabolites in a similar way and inactivate this type if basic host defense mechanism.

However, our data suggests the roles of HgSLP-1 and Rhg1 are not simple. Different isolates of SCN populations had variable copies of the *HgSLP-1* gene and the RNA-Seq data suggests that the mRNA encoded by the gene is alternatively spliced and forms containing intron sequences may be produced. Similarly, the Rhg-1 α-SNAP proteins show significant polymorphisms between and within SCN resistant and susceptible soybean lines [10,53]. The diversity of SCN SNARE-like proteins could reflect the nematodes attempt to adapt to different soybean α-SNAP proteins or other binding partners of HgSLP-1. Indeed, the apparent reduction in copy number of *HgSLP-1* from TN20 and other nematode populations may reflect a resistance evasion mechanism used by the nematode as several highly virulent SCN populations appear to have reduced copy numbers of the gene. However, if HgSLP-1 is important for establishing a feeding cell, one might expect that the lack of this protein may impair the growth and development of virulent SCN lacking the gene. While the lack of HgSLP-1 may play a role in evading host plant defense mechanisms, other potential virulence genes, such as the *HgBioB* or the gene near SNP 1533 are probably also required for virulence.

Overall, HgSLP-1 could play a role in SCN virulence, via its absence, but it could be involved with feeding cell formation if alterations of membrane fusion would be beneficial in this process. It seems plausible that HgSLP-1 may have other binding partners in the plant cell in addition to the *Rhg1* α-SNAP, but it will be important to localize HgSLP-1 at higher resolution in nematode feeding cells to validate its *in planta* role in nematode parasitism. It will be interesting in the future to test other α-SNAP variants to determine their binding kinetics to *HgSLP-1* in its various forms. It will also be important to follow up on the involvement in biotin in *Rhg1*-mediated resistance by further analysis of the biotin biosynthetic enzymes in the plant and nematode. Increasing our knowledge of how nematodes evade/suppress host plant resistance may lead to more durable SCN resistant plants or to improved methods of monitoring virulent nematode populations, which in turn will aid in the management of these damaging pathogens.

## Methods

### Development of SCN population for mapping and selection

Soybean cyst nematodes, populations TN10 and TN20 were grown by standard methods and cysts were harvested and purified as previously described [54]. SCN controlled mating was conducted using a modification of the method described in Dong and Opperman [33]. Briefly, 200 susceptible soybean seedlings (cv Essex unless otherwise stated) were germinated and planted into 50 ml sand filled falcon tubes, that previously had a hole drilled into the bottom and were fitted with an absorbent wick. The tubes containing the seedlings were placed in a tray of water so that the wick would keep the sand uniformly moist throughout the experiment. Soybean seedlings were inoculated with a single inbred SCN strain TN10 J2, which was allowed to parasitize the plant for three weeks. Male SCN were collected by inoculating susceptible plants with J2s from the inbred TN20 SCN strain, and then after a week washing the soil off the roots and placing the plants in a hydroponic culture to collect the males that emerged one week later. Soybean plants were inoculated with SCN TN20 one week after inoculation of the plants by TN10. To make the controlled cross, the soil was gently rinsed from the seedling inoculated with TN10 and visually inspected to identify SCN females. The plants containing the TN10 females were collected and replanted in sand and then inoculated with TN20 males. After a week, the F_1_ eggs were collected and used to re-inoculate a susceptible soybean plant and they were allowed to randomly mate for one generation. A sample of F_1_ J2s were also genotyped to verify they all were heterozygous (described below). The F_2_ eggs were collected and used to re-inoculate a susceptible plant for one more generation to produce the F_3_ SCN eggs, these again were used to infect susceptible plants, but some plants were placed into hydroponic culture to collect F_3_ unmated female nematodes for mapping, while others were allowed to mate to produce cysts for F_3_ derived single cyst lines used in the allelic imbalance analysis. Eighty four unmated females were harvested and frozen individually in 1.5 ml microcentrifuge tubes and stored at -80°C until use. The DNA extraction method described in [55] was used to extract the DNA from the unmated F3 female SCN.

For genotyping SCN F_1_ J2s, individual nematodes were placed in 0.2 ml PCR tubes and a one-step proteinase K DNA extraction method was used to liberate the nematode DNA [25]. The DNA was genotyped using a 2×TaqMan master-mix (Life Technologies) following manufacturer recommendations. The SNP assay, run on an Applied Biosystems (Foster City, CA) 7900HT Sequence Detection System under recommended settings using the following primers and probes: F-primer: GCGGCAGATTGAAGAAGCATTT, R-primer: GCACGGCACTGATCAGACA, Probe: FAM-CCTCTCCATGCGGACC-MGBNFQ, VIC-AGCCTCTCCATACGGACC-MGBNFQ. Standard PCR conditions were used for the TaqMan assays: 50C for 10 min, followed 95C for 10 minutes, then 40 cycles of 95C for 10 sec, and 60C for 1 min.

### Selection of SCN populations on resistant and susceptible plants

Single cyst SCN lines were allowed to grow for two generations on susceptible soybean (Essex) and then ten lines were harvested and equal amounts of eggs pooled. Two pools of ten SCN F_3_-derived lines were produced. Five SCN resistant (*Rhg1*) backcross 3 (BC3) and five susceptible BC3 soybean plants were inoculated with equal numbers of the pooled eggs and the nematodes were allowed to reproduce for one generation. One month later, the second pool of SCN eggs was used to inoculate a second set of five SCN resistant (*Rhg1*) backcross 3 (BC3) and five susceptible BC3 soybean plants. This set of plants served as a biological replicate for the allelic imbalance experiment.

For both experiments, the resulting cysts were harvested as described above, and approximately 50–100 cysts from each plant were placed into 1.5 ml microcentrifuge tubes, frozen in liquid nitrogen, pulverized with a steel pestle and then the DNA was extracted using a DNeasy tissue kit (Qiagen, Valencia CA) following the manufacturer’s instructions. The extracted genomic DNA (50 μl) was precipitated by adding 20 μg of yeast tRNA, 1 μl of pellet paint NF (EMD Millipore, Darmstadt Germany), 5 μl of 3 M Na acetate to the genomic DNA, followed by 2.5 volumes of cold 100% ethanol. The resulting DNA precipitate was collected by centrifugation and washed with 70% ethanol, and then air-dried. The DNA was amplified using a GenomiPhi kit following the manufacturer’s recommendations (GE Healthcare, Piscataway NJ). The resulting DNA was treated with ExoSAP-IT to remove primers and nucleotides following manufacturer’s instructions (Affymetrix). For all DNA samples the SCN DNA concentration was determined by SYBR green QPCR using 2 × SYBR green master-mix (Life Technologies, Grand Island NY), and primers GCCATTGGAGCGCCAGATGC and GGCTCATCGGCGGCACAA. Standard PCR conditions were used for the TaqMan assays: 50C for 10 min, followed 95C for 10 minutes, then 40 cycles of 95C for 10 sec, and 60C for 1 min, followed by a melting curve cycle. A standard curve of SCN DNA was known concentration was used to calculate the absolute concentration of the amplified SCN DNA.

### Allelic imbalance analysis

The SNP sequences were identified by comparing TN10 SCN cDNA sequences to SCN TN20 genomic DNA sequence. The TN10 cDNAs derived from J2 RNA (1,949,251 sequence reads) and egg RNA (3,080,637 sequence reads) were produced by a 454 GS FLX sequencer at the University of Illinois, Roy J. Carver Biotechnology Center and were assembled into contigs using the *de novo* assembly program in the CLC Genomics Work bench (CLCbio, Boston, MA). The TN10 contig sequences were concatenated into one million base lengths and used as reference sequences for the alignment of 577,902,089 TN20 genomic sequences (paired-end 25 nucleotide reads) generated on the SOLiD sequencing platform (SeqWright Inc, Houston TX). SNPs were identified in the alignments and selected if the aligned TN20 reads were different from the TN10 cDNA reference sequence, if the coverage was between 20 and 50 reads deep and if there were not other SNPs within 100 bp. Selected SNPs were also confirmed by alignment with TN10 genomic sequences, also produced on a SOLiD sequencing platform as described above. A list of 1,536 SNPs was sent to Illumina (San Diego, CA) to synthesize the GoldenGate genotyping oligonucleotides. The SCN DNA was diluted to a concentration of 100 ng/μl and 50 μl was sent for genotyping. The GoldenGate genotyping was conducted at the University of Illinois Functional Genomics Laboratory in the Roy J. Carver Biotechnology Center following standard protocols. After genotyping the SCN, genotypes and the theta values for each SNP and DNA sample were assigned using GenomeStudio V2011.1 (Illumina Inc). The SCN genetic linkage groups were produced by downloading the SNP data from the GenomeStudio program into Microsoft Excel where the SNPs were placed into phase with the parental nematode genotypes. The SCN genotypes, from 723 SNPs, were imported into MST Map [56] to produce linkage groups. The following settings were used: population type RIL3, population_name LG, distance_function kosambi, cut_off_p_value 1.0×10^−13^, no_map_dist 15.0, no_map_size 3, missing_threshold 0.25, estimation_before_clustering no, detect_bad_data yes, objective_function ML, number_of_loci 723, number_of_individual 84. The maps were drawn using the map draw macro in Microsoft Excel [57].

### Annotation of SCN scaffolds containing selected SNPs linked to virulence

The DNA sequence for each SNP that showed allelic imbalance was used to identify a SCN genomic sequence scaffold using BLASTN. The SCN scaffolds, build 1, were obtained from the JGI *Heterodera glycines* community genome sequencing project (http://genome.jgi.doe.gov/). The JGI SCN genome is from SCN inbred strain TN10. The sequence data was used to make and search a BLASTN database within the CLC Genomics Workbench. SCN scaffolds that matched the SNPs were annotated by performing a large-gap read mapping using SCN cDNA sequence derived from 454 and Illumina sequencing platforms. The mapped cDNAs were used to define the beginning and end of the expressed genes on the transcripts and intron sequences were removed to produce a final cDNA sequence. All expressed genes on the scaffold were compared to protein sequences in the databases via BLASTX. The cDNA sequence for HgSLP-1 was confirmed by PCR amplifying the full-length cDNA using primers flanking the open reading frame. To do this, TN10 RNA was extracted and converted to cDNA as described in [25]. The cDNA was amplified using a proof-reading thermostable DNA polymerase and then cloned into pCR2.1 plasmid vector. The DNA sequenced was determined at the University of Illinois Roy J. Carver Biotechnology Center. To identify DNA polymorphisms in the candidate virulence genes, genomic DNA sequence derived from SCN strain TN20 was mapped to each scaffold using the CLC Genomics Workbench and SNPs were identified using the quality-based variant detection program. The coiled-coil domain was identified using the program COILS on the ExPASy web site [58]. The t-SNARE domain was detected and the multiple sequence alignment was produced using NCBI’s conserved domain database [59], but the conserved, zero-layer polar amino acid residue with the flanking hydrophobic amino acid heptad repeats were detected using hydrophobic cluster analysis [60]. The signal peptide was predicted using the TargetP 1.1 server [61], also found on the ExPASy web site.

### Copy number analysis for HgSLP-1

SCN genomic DNA was extracted as described above for SCN strain TN10, TN20, OP20, OP25 and OP50. Two TaqMan assays were used to measure the copy number of the *HgSLP-1* gene relative to a control gene, *HgFAR-1*. Standard PCR conditions were used for the TaqMan assays: 50C for 10 min, followed 95C for 10 minutes, then 40 cycles of 95C for 10 sec, and 60C for 1 min. For each DNA type, triplicate PCR reactions were conducted and the entire experiment was conducted twice with similar results. The data was compared using the ΔCt method [62], where ΔCt = Ct^target^-Ct^control gene^ and the fold difference (FD) between the *HgSLP-1* target gene and the HgFAR-1 control gene was calculated using the equation, FD = 2^-ΔCt^. The fold differences were normalized to OP25 since there was nearly no difference in ΔCt for this SCN population. *HgFAR-1* was known to be of consistent copy number Craig et al. (2008). The primer and probes for detection of HgFAR-1 were as follows: F-primer AGGTGACCAAATTCTACC, R-primer GGGTGTCCATTTATTTGC, Probe FAM-CTGACCGAGGATGGACAA-MGBNFQ. The HgSLP-1 TaqMan primers and probes detected sequences in the second exon of HgSLP-1, the following oligonucleotides were used: F-primer, CGAGATGAAATGAACCAAA R-primer, GAGTCGTTTGTCCATTTG Probe FAM-AACACGAGATTGGAC -MGBNFQ.

### Localization of HgSLP-1 protein in SCN-infected soybean roots

Antibodies to HgSLP-1 were generated from a synthetic peptide (CRHLFESGEASETAS) in rabbits and affinity purified by GenScript (Piscataway, NJ). Susceptible soybean seedlings (Essex) were inoculated with inbred SCN strain TN10 eggs, which were allowed to hatch and infect the soybean roots for 5 days to generate a mixed age population of nematodes. Root sections containing SCN infection sites were dissected and 0.5 cm long root segments were placed in ice-cold FAA fixative (50% ethanol, 5% acetic acid, 10% formalin). The nematodes and roots were microwave-fixed and embedded in paraffin [15]. Immunolocalization of HgSLP-1 was conducted by sectioning (10 μm) the paraffin embedded roots and mounting them on Probe-on-Plus slides (Fisher Scientific, Pittsburgh PA) over night at 42°C. The slides were soaked twice in xylene for 5 minutes, and then incubated in 100% acetone for 5 minutes. After repeating the acetone incubation the slides were hydrated by soaking for 5 minutes each in 95%, then 85%, 70%, and 50% acetone. Finally they were soaked in distilled H_2_O and phosphate buffered saline (PBS: 137mM NaCl, 2.7 mM KCl, 10mM Na2HPO4, KH2PO4 pH 7.4) for 5 minutes each. The nematodes were permeablized by incubating the nematodes in PBS containing 4 μg/ml proteinase K for 30 minutes. The proteinase K was inactivated by soaking the slides 5 minutes in PBS with 0.2% glycine, then in PBS with 1mM phenylmethanesulfonylfluoride, followed by a 5 minute treatment in PBS. The slides were blocked with 10% goat serum in PBS for 30 minutes, and then washed with PBS with 0.1% bovine serum albumin (BSA) for 5 minutes. The slides were incubated for two hours with the HgSLP-1 primary antibody or pre-immune serum as a negative control (1:250 dilution) in PBS with 0.1% BSA, then washed 3 times, 10 minutes each wash, in PBS with 0.1% BSA. Next, the slides were incubated with the secondary antibody, goat-anti-rabbit Oregon green 488 (Life Technologies) in PBS with 0.1% BSA (1:250 dilution) and incubated for 2 hours. Finally, the slides were washed 3 times (10 minutes each wash) in PBS with 0.1% BSA. The slides were washed once with water and mounted with a coverslip using Prolong anti-fade and allowed to dry overnight. The sections were observed on a Zeiss Axioscope 2 florescence microscope and digital images were captured with a Zeiss Axiocam. These experiments were conducted three times with similar results.

### Production of α-SNAP antibodies

Total RNA from soybean cv Forrest was converted to cDNA and used as a template to PCR amplify α-SNAP (Glyma18g02590) accession number LOC100814639 using primers *Xma*I-SNAP-F: AAACCCGGGAATGGCCGATCAGTTATCGAAGG and *Xho*I-SNAP- R: AAAACTCGAGTCAAGTAATAACCTCATACTCC. The resulting PCR product was cloned between *Xma*I and *Xho*I cloning sites in the pGEX-5x-1 vector that adds a GST-tag (pGEX-5x-1-SNAP-GST). The construct was confirmed to be correct by sequencing (Genewiz, South Plainfield, NJ). The plasmid was transformed into *E*. *coli* BL21 and sent to Rockland Immunochemicals (Gilbertsville, PA) where the company produced and purified the protein and then injected it into rabbits. The SNAP polyclonal antibody was affinity purified and cross adsorbed to remove antibodies that might bind to the GST tag.

### Characterization of HgSLP-1 by co-expression/co-purification

The HgSLP-1 protein sequence derived from SCN strain TN10 and the susceptible allele of the soybean α-SNAP were used to synthesize the coding region using DNA Strings (Life Technologies). HgSLP-1 cDNA was PCR amplified using primer HgSLP1F-*Bgl*II: GGTCTTGAGCGGATATCTTAACCGG and primer HgSLP1R-*Eco*RV: CTCGACATCTCAGATCTATGGCACC which had had terminal start-*Bgl*II and *Eco*RV-end sites for cloning. The cDNA was PCR amplified using CloneAmp HiFi PCR premix (Clontech, Mountain View CA) and 10 pmoles of each primer using 98C, 10 sec and 25 cycles of 98C for 10 sec, 60C for 15 sec, 72C for 10 sec followed by a 72C for 2 min. The PCR products were gel purified and the cDNA insert was digested with the restriction enzymes *Bgl*II and *Eco*RV and ligated into the second expression position of the vector pCDF Duet-1 (Novagen, San Diego, CA) digested with the same enzymes. The pCDF Duet-1-HgSNP-1 vector was then transformed into *E*. *coli* Top10 cells. Selection was performed on spectinomycin (50 mg/ml) LB agar plates. The resulting plasmid containing *HgSLP-1* was verified for accuracy by sequencing the nematode gene at the University of Illinois Roy J. Carver Biotechnology Center. Later the *HgSLP-1* plasmid was transformed into JM109DE3 *E*. *coli* and used as a negative control that expresses only the HgSLP-1 protein.

The signal peptide was removed from the *HgSLP-1* gene in the pCDF-Duet-1 vector by PCR amplifying the plasmid with primers HgSLP-1deltaspF: GAAAAAGCAGCACCGAATGC and HgSLP-1deltaspR: CGGTGCTGCTTTTTCCATAGATCTGCCATATGTATATCTCCT. The PCR was conducted using CloneAmp HiFi PCR premix as described above, except that the extension time was 30 seconds. The resulting PCR product was treated with cloning enhancer, the plasmid was circularized using the In-Fusion HD enzyme premix and was transformed into *E*. *coli* Stellar competent cells following the manufacturers protocol (Clontech). The resulting plasmid containing *HgSLP-1* missing the signal peptide was verified for accuracy by sequencing the nematode gene as described above.

The cDNA of the soybean α*-SNAP* was synthesized using DNA Strings (Life Technologies). The cDNA was PCR amplified using primers soysnapF-*Eco*RI: TCGCGAATGCGAATTCATGGCCGAT and soysnapR-*Sal*I: CGTCGAGCATGTCGACTCAATGGTG. The cDNA was digested with *Eco*RI and *Sal*I and ligated into the first expression position of pCDF Duet-1-HgSLP-1. The plasmid was then transformed into JM109DE3 *E*. *coli* that contains the T7 RNA polymerase gene and allows isopropyl-beta-D-thiogalactopyranoside (IPTG) induction of the lac-T7 promoters on pCDF-Duet-1. For all protein expression experiments, the *E*. *coli* containing the construct was grown at 37C until OD600nm was 0.4, then it was induced with 1 mM IPTG for 4 hours at 30C. The induced *E*. *coli* cells were collected by centrifugation, suspended in50 mM sodium phosphate (pH 7.4), 150 mM NaCl containing 1x Halt Protease Inhibitor cocktail, lacking EDTA (Thermo Scientific) and lysed using a B-PER bacterial protein extraction kit (Fisher Scientific) following the manufacturer’s instructions. The protein concentration of the resulting *E*. *coli* protein extract was determined using the Pierce BCA protein assay kit (Fisher Scientific). Equal concentrations of proteins from the negative control and experimental samples were purified using Dynabeads His-Tag Isolation kit (Life Technologies) following manufacture’s protocol. The eluted proteins were run on a Mini-Protean TGX SDS-PAGE 4–20% gradient gel (BioRad, Hercules CA) and the proteins were transferred to a nitrocellulose membrane using an iBlot dry blotting system (Life Technologies). The protein blot was incubated with blocking buffer (0.1M maleic acid, 0.15M NaCl, 1% BSA, 0.3% Triton X-100) for one hour. The primary polyclonal antibodies, anti-HgSLP-1 or anti- α-SNAP, were diluted 1:5000 in blocking buffer and incubated with the blot for 30 minutes and were then washed with blocking buffer three time for 10 minutes each. The secondary antibody, goat anti-rabbit alkaline phosphatase conjugate, was also used at a 1:5000 dilution in blocking buffer and was incubated and washed as described above. The secondary antibody was detected using Western Blue Stabilized substrate for alkaline phosphatase (Promega, Madison WI) and the reaction was stopped using TE (10mM Tris-HCL, 1mM EDTA) after approximately 20 minutes of development at room temperature. The experiment was repeated with similar results.

The total *E*. *coli* protein extracts expressing HgSLP-1 alone or both HgSLP-1 and -α SNAP were analyzed by gel filtration chromatography. The gel filtration column was 50 cm tall and had a diameter of 1.8cm. The column was packed with Superdex 75 prep grade resin (Amersham Biosciences) and a BioRad Econogradient pump. The protein fractions were collected on a BioRad model 2110 fraction collector. The column was equilibrated using 50 mM sodium phosphate (pH 7.4), 150 mM NaCl buffer and then calibrated using a LMW gel filtration calibration kit (Amersham Biosciences). Blue dextran 2000 was used to determine the void volume of the column and ribonuclease A (13.7 kDa), chymotrypsinogen A (25.0 kDa), ovalbumin (43.0kDa), albumin (67.0kDa) were used in the column calibration. For each column run, the flow rate of the column of 0.25ml/minute and each fraction was collected for two minutes. Before each sample was added, 200 ul of blue dextran 2000 was added to the column, allowed to run into the column for one minute, then 200 ul of protein extract was added (either HgSLP-1 or the extract containing both HgSLP-1 and α-SNAP). The extracts were the same ones used in the co-purification experiments described above. The fractions obtained were tested for the presence of HgSLP-1 using protein dot blots. Briefly, 1600 ul of cold acetone was added to 400 ul of each fraction, the proteins were allowed to precipitate on ice for 30 minutes, then they were centrifuged and the acetone was removed from the protein pellet. The proteins were dried in a Speedvac and then suspended in 10 ul of 1x SDS PAGE buffer and boiled for 5 minutes. 2 ul of the protein fraction was spotted onto nitrocellulose, dried, and then the HgSLP-1 was detected as described above. The resulting spots were quantified using NIH image and the resulting intensities were plotted using Microsoft Excel. The experiment was repeated with similar results.

### DNA and RNA sequence data

*HgSLP-1* gene sequence is deposited in GenBank accession number KM575849. All next-generation DNA sequence data used in this project is deposited in the NCBI BioProject 680464 titled, “Heterodera glycines genome sequencing”

Genomic sequence: SCN strain TN20 SOLiD (2x25base 3kb mate pair) 577,902, 089 reads

Genomic sequence: SCN strain TN10 SOLiD (50 base) 270,363,891 reads

RNAseq: SCN strain TN10 454: J2 RNA (1,949,251 sequence reads) and egg RNA (3,080,637 sequence reads) 200-500base.

RNAseq: SCN strain TN10 Illumina (2x75base paired end) 263,530,527 reads

RNAseq: SCN strain TN10 Illumina (2x100base paired end) 365,288,386 reads

SCN SNPs: 1536 SNP and flacking sequence

DNA sequences for scaffolds: 385, 1924 and 20.

## Supporting Information

S1 Table SCN genotypes for SNP markers found in linkage groups 1 and 2 of the SCN genetic map. Parental genotypes are denoted by A or B and heterozygous nematodes are represented by an X.
(TXT)
